# 

**Published:** 1923-03

**Authors:** 


*The following donations have been received since the publication of the List in September.*

*March*, 1923.

| | |
|---|---|
| Mr. Hey Groves (1) .. .. .. .. | 7 volumes. |
| Dr. R. G. Poole Lansdown (2) .. .. | 1 volume. |
| Medical Research Council (3) .. .. .. | 1 ,, |
| Dr. J. E. Shaw (4) .. .. .. .. | 2 volumes. |
| J. Wright & Sons Ltd. (5) .. .. .. | 1 volume. |

Unbound periodicals have been received from Mr. Hey Groves and Dr. Shaw.

### THE ONE HUNDRED AND SEVENTH LIST OF BOOKS.

---

The figures in round brackets refer to the figures after the names of the donors, and show by whom the volumes were presented. The books to which no such figures are attached have either been bought from the Library Fund or received through the *Journal.*

**Adami, J. G.** .. .. *Charles White and Puerperal Fever* .. .. 1922
**Allbutt, Sir T. C.** .. *Notes on the composition of Scientific Papers* [3rd Ed.] .. 1923
**Bainbridge, W. S.** *Le Problème du Cancer* .. .. .. .. (1) 1922
**Beesly and T. B. Johnston, L.** *Surgical Anatomy* .. .. 2nd Ed. 1922
**Bell, Sir C.** .. .. *Anatomy and Physiology of Expression* (2) 3rd Ed. 1844
**Blomfield, J.** .. *Anæsthetics* .. .. .. .. .. .. .. .. 1922
**Brown, W. L.** .. *The Sympathetic Nervous System in Disease* 2nd Ed. [1923]
**Brunet, J. C.** .. *Manuel du Libraire.* 5 vols... 4me Éd. 1838–45
**Burrows, H.** .. .. *Mistakes and Accidents in Surgery* .. .. .. 1923
**Carter, H.** (Ed.) .. *The Church and the Drink Evil* .. .. .. [1922]
*Cunningham's Text-book of Anatomy* (Ed. by A. Robinson) 5th Ed. [1922]
**Daniels and H. B. Newman, C. W.** *Laboratory Studies in Tropical Medicine* .. .. .. .. .. 5th Ed. 1923
**Friel, A. R.** .. .. *Electric Ionization* .. .. .. [2nd Ed.] 1922
**Fuchs, E.** .. .. *Text-Book of Ophthalmology* (Tr. by A. Duane) 7th Ed. [1923]
**Fuller, A. W.** .. *Anæmia* .. .. .. .. .. .. 1923
**Gamberini, C.** .. *Sull' Epilessia postraumatica* .. .. .. (1) 1921
*Gouernayle of Helthe, The* (Facsimile Reprint) .. .. .. .. (4) 1858
**Hadfield, C. F.** .. *Practical Anæsthetics* .. .. .. .. .. .. 1923
**Ham, R. B.** .. .. *Handbook of Sanitary Law* (Ed. by H. R. Kenwood) .. .. .. .. .. 9th Ed. 1923
**Hauck, L.** .. .. *Die Behandlung der Geschlechtskrankheiten* (5) 1922
**Hewitt, Sir F. W.** *Anæsthetics* (Ed. by H. Robinson) 5th Ed. 1922
**Hinton, J.** .. .. *The Place of the Physician* .. .. .. (4) 1874
**Johnston, L. Beesly and T. B.** *Surgical Anatomy* .. .. 2nd Ed. 1922
**Keith, A.** .. .. *The Engines of the Human Body* .. .. .. 1919
**Korenchevsky, V.** .. *Aetiology and Pathology of Rickets* .. (3) 1922
**Lindsay, J. A.** .. *Medical Axioms and Aphorisms* .. .. .. 1923
**McKay, W. J. S.** *Lawson Tait: his Life and Work* .. .. 1922
**Myers, B.** .. .. *Diseases of Children* .. .. .. .. .. .. 1922
**Nesfield, V.** .. .. *Ophthalmic Surgery* .. .. .. .. .. .. 1922

| | | | |
|---|---|---|---|
| **Newman, C. W. Daniels and H. B.** | *Laboratory Studies in Tropical Medicine* .. .. .. .. .. | 5th Ed. | 1923 |
| **Price, F. W.** (Ed.) | *Practice of Medicine* .. .. .. .. .. | | 1922 |
| **Rolleston, Sir H.** | *Some Medical Aspects of Old Age* .. .. .. | | 1922 |
| **Romanis, W. H. C.** | *Surgical Diagnosis* .. .. .. .. .. .. | (1) | 1923 |
| **Short, A. R.** (Ed.) | *Index of Prognosis* .. .. .. .. | 3rd Ed. | 1922 |
| **Short, A. R.** .. | *New Physiology in Surgical and General Practice* .. .. .. .. .. .. | 5th Ed. | 1922 |
| **Tidy, H. L.** .. .. | *A Synopsis of Medicine* .. .. .. | 3rd Ed. | 1923 |
| **Voronoff, S.** .. .. | *Greffes testiculaires* .. .. .. .. .. .. .. | | 1923 |
| **White, J. R.** .. | *Clinical Examination of Surgical Cases* | (1) | 1922 |
| **Whitla, Sir W.** .. | *Elements of Pharmacy* .. .. .. | 11th Ed. | 1923 |

TRANSACTIONS, REPORTS, JOURNALS, ETC.

| | | |
|---|---|---|
| American Ophthalmological Society, Tiansactions of the | Vol. XX. | 1922 |
| American Otological Society, Transactions of the | Vol. XVI., Pt. 1 | 1922 |
| British Medical Journal, The .. .. .. .. .. | Vol. II. for | 1922 |
| College of Physicians of Philadelphia, Transactions of the | Vol. XLIII. | 1921 |
| Hospitals— | | |
| St. Bartholomew's Hospital Repoits .. .. .. .. .. | Vol. LV. | 1922 |
| Jahresbericht über die gesamte Chirurgie .. .. .. .. | (1) | 1922 |
| Lancet, The .. .. .. .. .. .. .. .. .. .. | Vol. II. for | 1922 |
| London Dermatological Society, Transactions of the .. .. .. | | 1922 |
| Merck's Jahresbericht .. .. .. .. .. .. .. | XXXV. Jahıg. | 1922 |
| Ophthalmological Transactions .. .. .. .. .. .. | Vol. XLII. | 1922 |
| Royal Society of Medicine, Proceedings of the .. .. | Vol. XV. | 1922 |

---

### Local Medical Notes.

University of Bristol.—Undergraduates of the University have recently passed the following examinations :—

M.B., Ch.B. Bristol.—*Second Examination, Part I.:* D. E. C. Andrew, J. C. Batt, A. M. Critchley, T. F. Hewer, R. Jackson, E. R. Wide. *Elementary Anatomy only:* B. J. Boulton, J. M. Camps-Campins, M. Srisvasti. *Part II.:* H. M. Aldwinckle, E. C. Bernard, W. P. Brinckman, M. E. Drew, G. L. Feneley, F. R. Gedye, A. P. Gorham, M. B. Harvey, T. P. Lalonde, E. May, C. B. Perry, L. B. Phillips, A. A. B. Vincent, T. M. White. *Final Examination: Pass (with second*

*class honours)* : F. J. Hector, M. G. Hughes, A. J. Keevill, D. M. Pullen. *Part II.* (*completing examination*) : B. A. Crook, J. M. Evans, F. M. Jones, J. A. L. Roberts, H. L. Shepherd, H. J. H. Spreadbury, F. K. Wilson. *Part I.* (*including Forensic Medicine and Toxicology*) : S. H. Blacker, A. P. Bodman, E. G. Bradbeer, G. B. Bush, W. L. Cossham, M. F. Dalton, F. R. Edbrooke, N. J. England, H. M. Golding, W. A. Gornall, J. L. Griffin, R. C. Hatcher, A. G. Heron, D. E. Joscelyne, P. C. Joscelyne, E. F. King, A. H. Marshall, J. R. Nicholson-Lailey, R. A. Sammons, A. E. Sherwell, H. Taylor, R. E. Whitby, C. F. Wilson. *Part I. only* : N. J. Bown, H. M. Dixon, L. Dunn, P. G. Evans.

L.D.S. Bristol.—*Second Examination* : W. H. Phillips, B. F. Taylor, L. H. Challenger, R. Lonnon, J. F. Rigg, J. R. Ritblat. *In Physiology and Histology and Anatomy only* : G. C. Brooks. *In Lental Anatomy only*: A. W. Cawsey, C. S. Cossham, R. G. Downes, D. Griffiths, C. H. Hill, H. F. D. Lane, E. W. Mogg, S. Phillips, B. F. Robinson, A. M. Thoseby, C. S. Wilde, C. P. Winstone. *Third Examination* : G. C. Brooks, C. H. Hill, E. W. Mogg. *Final Examination* : F. J. Farr,* H. W. Pidgeon,* G. G. Royal,* C. G. Saxon,* C. R. Woods.*

D.P.H.—Zako Khalid.

Conjoint Examining Board of England.—*Second Examination* : C. C. Chesterman. *Final Examination* : B. A. Crook,* J. M. Evans,* E. C. K. Kenderdine,* J. A. L. Roberts,* H. J. H. Spreadbury.*

L.D.S., R.C.S.—S. G. House.*

L.M.S.S.A.—*Midwifery, Medicine, Forensic Medicine* : C. S. Laurence.

* Denotes qualification.

